# Thousands of RAD-seq Loci Fully Resolve the Phylogeny of the Highly Disjunct Arctic-Alpine Genus *Diapensia* (Diapensiaceae)

**DOI:** 10.1371/journal.pone.0140175

**Published:** 2015-10-08

**Authors:** Yan Hou, Michael D. Nowak, Virginia Mirré, Charlotte S. Bjorå, Christian Brochmann, Magnus Popp

**Affiliations:** 1 Natural History Museum, University of Oslo, Oslo, Norway; 2 Science for Life Laboratory, Stockholm University, Solna, Sweden; CNRS / Université Joseph-Fourier, FRANCE

## Abstract

Restriction-site associated DNA sequencing (RAD-seq) has recently become an important method to generate genome-wide molecular data for species delimitation, phylogeography, and population genetic studies. However, very few empirical studies have so far tested its applicability in phylogenetic reconstruction. The alpine-arctic genus *Diapensia* was selected to study the origin of the disjunction between the Arctic and the Himalayan-Hengduan Mountains (HHM). However, a previous phylogenetic analysis based on one nuclear and four plastid DNA regions failed to resolve the oldest divergences in *Diapensia* as well as the relationship between the two HHM species. Here we reconstruct a fully resolved phylogeny of *Diapensia* and address the conflict between the currently accepted taxonomy and the gene trees in the HHM species using RAD-seq. Based on a data set containing 2,650 loci selected to maximize the number of parsimony informative sites and allowing for a high level of missing data (51%), the phylogeny of *Diapensia* was fully resolved and each of the four species was reciprocally monophyletic. Whereas the arctic *D*. *lapponica* was inferred as sister to the HHM clade in the previous study, the RAD-seq data resolved the two arctic species as sisters to the HHM clade. Similar relationships were inferred from a differently filtered data set with far fewer loci (114) and less missing data (21%), but with lower support and with one of the two HHM species as non-monophyletic. Bayesian concordance analysis and Patterson’s D-statistic tests suggested that admixture has occurred between the two HHM species.

## Introduction

The selection of appropriate and sufficient molecular markers is fundamental to phylogenetic reconstruction, and the emergence of next-generation sequencing (NGS) technologies provides numerous possibilities for improvement. Traditional Sanger sequencing [1] of plastid DNA (pDNA) markers and nuclear ribosomal markers such as the internal transcribed spacer (ITS) have been widely applied to reconstruct plant phylogenies at the species and genus level. However, due to maternal inheritance of plastids, phylogenies constructed based on pDNA data are limited in their capacity to reflect the evolutionary history of a lineage. Multi-copy nuclear markers such as ITS can mislead phylogenetic inference because of concerted evolution [2]. Low-copy nuclear genes have been successfully applied in interspecific phylogenetic inference [3]. However, only a limited number of low-copy nuclear genes have been used in most empirical studies, because searching for phylogenetically informative low-copy nuclear markers with traditional Sanger sequencing is costly and laborious work. Increasing the number of unlinked molecular markers in phylogenetic analyses can dramatically improve the accuracy of phylogenetic reconstruction [4]. In this way, NGS technologies offer an efficient and cost-effective approach to sequence millions of nucleotides for phylogenetic inference.

Restriction-site associated DNA sequencing (RAD-seq) has been recognized as an economical and efficient method for discovering genome-wide genetic markers [5–7]. This approach uses NGS technology to sequence short DNA fragments adjacent to restriction enzyme recognition sites in a genome. One of the main advantages of RAD-seq is that it does not require previously developed genomic resources, such as genome or transcriptome assemblies, making it particularly useful in non-model species [8–11].

The RAD-seq method has been successfully applied in studies of intraspecific genetic diversity and phylogeographic history [12–15]. Analyses of empirical and simulated RAD-seq data have shown it to be a powerful tool for inferring phylogenetic relationships at the interspecific scale as well [16–20]. The primary challenge in applying RAD-seq to reconstructing interspecific phylogenies lies in confidently identifying and assembling orthologous loci amongst the relatively short (i.e. usually 100 to 200 bp), usually non-coding sequence fragments produced with this method [16]. This problem stems from the fact that the number of restriction sites that are conserved among taxa is expected to decrease with increased time since divergence, implying that RAD-seq data may be of limited use in more ancient clades [17]. Nevertheless, empirical RAD-seq data has been successfully used to resolve the phylogeny of American oaks, which is a 23–33 million years old (Ma) clade [19], and simulated RAD-seq data has been used to accurately estimate the phylogeny of a hypothetical clade that shared a common ancestor 60 Ma [16].

As the number of molecular markers increases, the process of inferring phylogenies also faces new challenges. Individual loci may have different evolutionary histories due to incomplete lineage sorting, gene duplication or loss, and processes of admixture such as hybridization and introgression [21]. The RAD-seq method may be a promising tool for phylogenetic inference under such circumstances. With RAD-seq data sets consisting of over 3 million base pairs, the phylogenetic relationships among the sympatric Lake Victoria cichlid species were successfully resolved, despite the fact that this group is characterized by recent adaptive radiation, incomplete lineage sorting and ongoing hybridization [22]. RAD-seq data has also been used to detect current or historical introgression, using the Patterson’s D-statistic test [18, 23].

**Fig 1:**
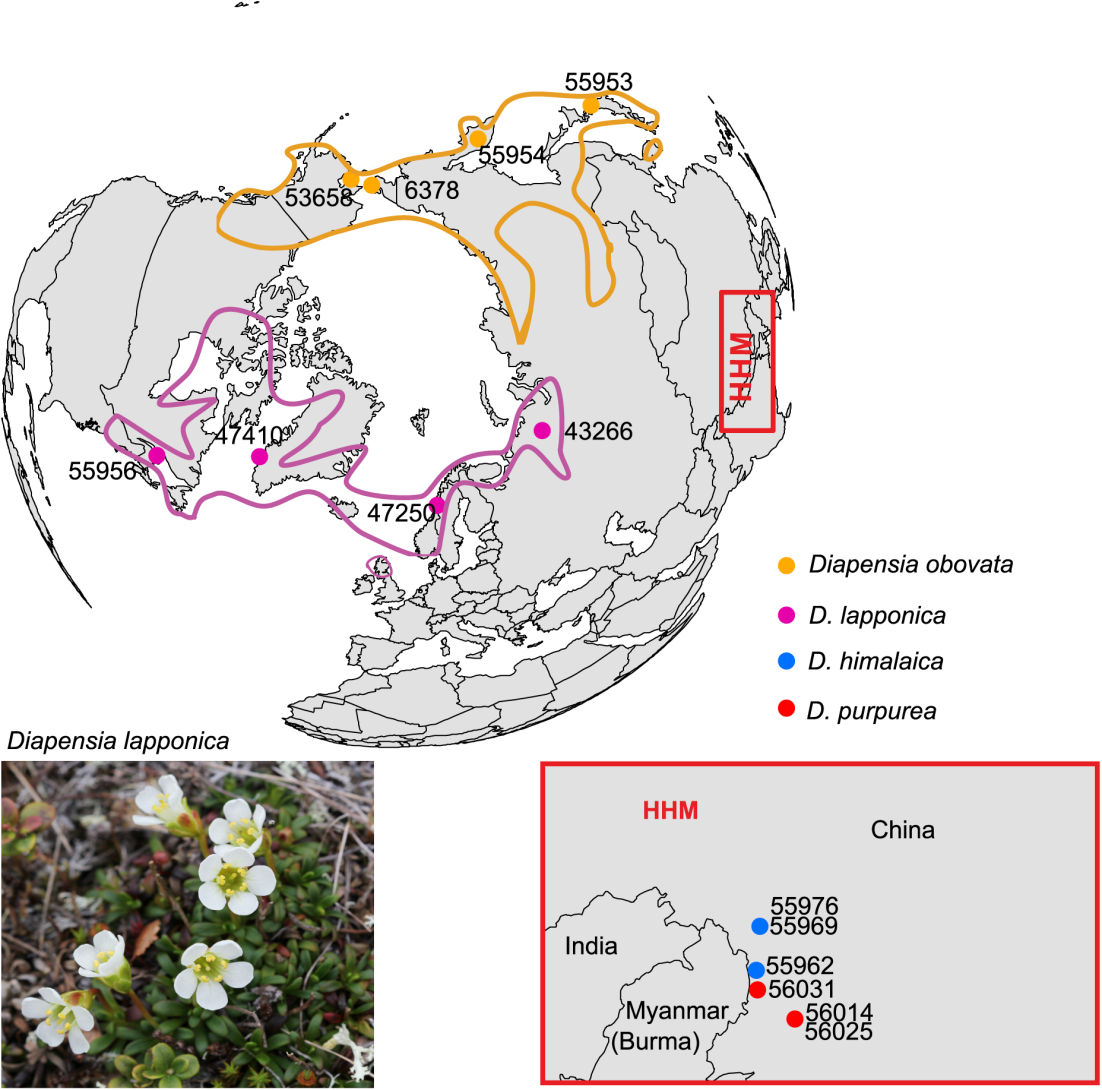
Total geographical ranges (lines) and sampling sites (dots) of *Diapensia*. HHM: Himalayan-Hengduan Mountains. Sample IDs refer to Table 1. The ranges of *D*. *lapponica* and *D*. *obovata* are redrawn after Hultén & Fries [44]. The photo of *D*. *lapponica* was attributed by Alinja (https://commons.wikimedia.org/wiki/File:Diapensia_lapponica_Kilpisj%C3%A4rvi_2012-07.jpg#/media/File:Diapensia_lapponica_Kilpisj%C3%A4rvi_2012-07.jpg)

*Diapensia* is a genus of arctic-alpine subshrubs consisting of five species. *Diapensia lapponica* L. is broadly amphi-Atlantic and *D*. *obovata* (F.Schmidt) Nakai is broadly amphi-Beringian with southwards extension into Central Asia [24, 25]. Three species, *D*. *himalaica* J.D.Hooker & Thomson, *D*. *purpurea* Diels, and *D*. *wardii* W.E.Evans, are endemic to the Himalayan-Hengduan Mountains (HHM; [26]). The origin of the Arctic and HHM disjunction (Fig 1) and the phylogeny of the genus was addressed by Hou *et al*. [27] using four plastid DNA regions and ITS. Three major clades were identified and estimated to be of Late Miocene origin. However, the relationship between the three main lineages remained uncertain, and multiple accessions of the two HHM species were mixed.

In this study, we apply RAD-seq data to resolve the phylogenetic relationships in the disjunct arctic-alpine genus *Diapensia*. To test whether admixture has occurred between the two HHM species previously identified as paraphyletic with respect to each other, we applied Bayesian concordance analyses and Patterson’s D-statistic tests to the RAD-seq data matrix.

## Materials and Methods

### Taxon sampling, DNA extraction, RAD-seq library preparation and sequencing

Our data set consists of 18 samples representing four of the five extant species of *Diapensia* (Fig 1; S1 Table) and the two outgroup species *Shortia uniflora* Maxim. and *Schizocodon soldanelloides* Siebold & Zucc. (S1 Table). Vouchers are deposited in the Herbarium of the Natural History Museum in Oslo (O) or the Kyoto University Museum (KYO; S1 Table). Total genomic DNA was extracted using the DNeasy Plant Mini Kit (Qiagen, Valencia, CA, USA) following the manufacturer’s standard protocol. The narrow Tibetan endemic *Diapensia wardii* is not included in this study because we did not manage to collect this species in the field, and we were unable to extract DNA of sufficient quality from the single available herbarium specimen of this species despite several attempts.

RAD-seq libraries for Illumina paired-end sequencing were prepared following Etter *et al*. [5] with some minor modifications (S1 Text). Briefly, genomic DNA from each sample was digested with the high-fidelity restriction enzyme *Sbf*I (New England Biolabs, Ipswich, MA, USA), and Illumina sequencing adaptors containing sample-specific barcode sequences were ligated to the fragmented DNA. The barcode sequences contain five nucleotides with at least three nucleotide differences between each barcode sequence. The libraries were multiplexed 16× and sheared using a Bioruptor (Diagenode, Denville, NJ, USA), and fragments between 250 and 500 bp in length were selected by gel extraction using the MinElute Gel Extraction Kit (Qiagen). Paired-end sequencing of the multiplexed libraries was conducted on an Illumina HiSeq 2000 instrument at the Norwegian Sequencing Centre using 101 cycles.

### Processing and clustering RAD-seq data

Following standard Illumina processing and quality filtering, duplicate reads resulting from PCR amplification were discarded using the program clone_filter implemented in the Stacks v. 1.20 software [28]. The resulting forward reads were de-multiplexed, quality filtered and *de novo* clustered using pyRAD v. 2.12, a pipeline optimized to produce aligned orthologous RAD-seq loci from NGS raw reads across distantly related taxa [29]. We only present the results based on RAD-seq forward reads, which are deposited as a BioProject in the Sequence Read Archive database with accession number SRP062066.

**Fig 2:**
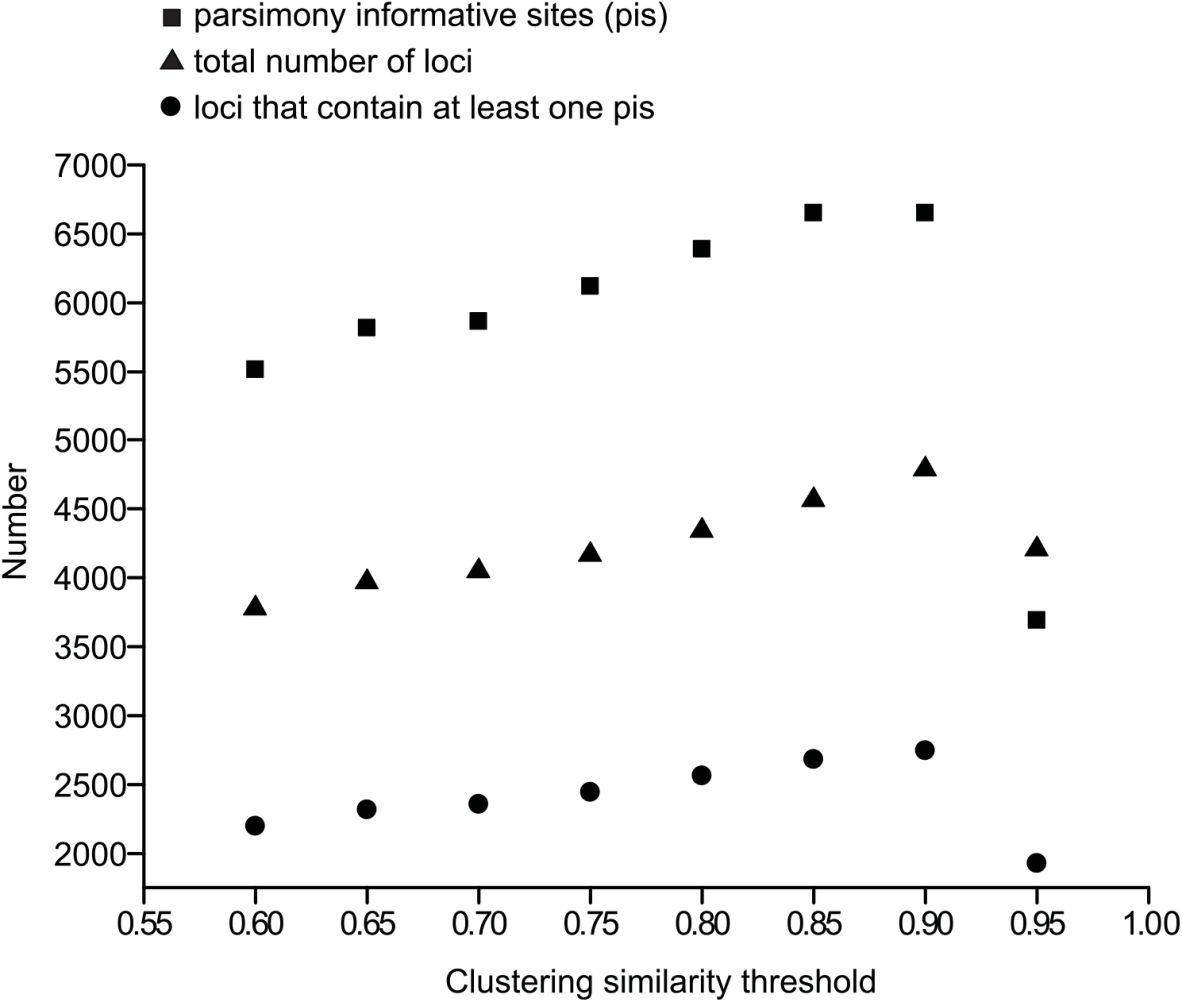
Correlation between three important statistics in RAD-seq data sets and clustering similarity thresholds. Square: number of parsimony informative sites; triangle: total number of loci; and dots: number of loci that contain at least one parsimony informative site.

The de-multiplexing was performed based on the sample-specific barcode sequences, allowing for one mismatch in the barcode sequence. Base calls with a Phred quality score under 20 were converted to Ns, and reads containing more than 4 Ns were discarded. Once the adapter sequences, barcodes, and restriction site sequences were removed, the final length of the forward reads was 90 bases. For within-sample clustering a minimum coverage cutoff of 2× was employed. When clustering across samples, loci with a heterozygous site that was shared by more than two samples were discarded as putative paralogs, and loci containing more than 10 SNPs were discarded. The same clustering threshold was used for both within- and across-sample clustering [29]. We tested a range of clustering thresholds (60–95% in 5% increments) and minimum number of samples (m) that had to be shared by each locus in the final aligned data matrix (i.e. m = 4 or 14). A final clustering threshold of 90% was chosen to construct data sets for further analysis because this value provided the highest number of loci and parsimony informative sites (Fig 2). We only present the results based on two data sets with a minimum number of 4 or 14 samples required per locus, herein referred to as ‘m4’ and ‘m14’ data set, respectively. Loci that did not contain any parsimony informative site were excluded from the pyRAD output data, and the last 5 bases of each locus were trimmed because the base-call quality was found to drop precipitously in this region. The resulting RAD-seq loci were blasted against the NCBI remote BLAST nucleotide database, using the program blastn in BLAST+ 2.2.29 (ftp://ftp.ncbi.nih.gov/blast/executables/LATEST/) with default settings and an “*E*-value” significance threshold of 1 × 10^−4^. Loci that had hits to any sequences that did not originate from green plants were discarded for further analysis.

### Phylogenetic analyses

The above steps resulted in two aligned and concatenated RAD-seq data matrices, with heterozygous sites coded using IUPAC standard ambiguity codes. Maximum likelihood trees were inferred for the m4 and m14 data matrices using RAxML v. 7.8.3 [30] with random starting trees and the GTR + G nucleotide substitution model, and support was estimated by performing 1000 bootstrap replicates.

We also conducted Bayesian phylogenetic inference based on the two data matrix using MrBayes v. 3.2.1 [31]. The software jModelTest v. 2.1.7 [32] was used to select the best-fit substitution model for each locus, and the results are presented in S2 Table. The loci sharing the same best-fit model were defined as one partition. For each data matrix, two independent runs were performed with random starting trees and the best-fit substitution model for each data partition. Each run was performed with four chains (one cold chain and three hot chains) for 10 million generations and with sampling every 1000 generations. Default priors were used in all analyses. The convergence of parameters among runs was evaluated visually using Tracer v. 1.6 [33]. The consensus trees and Bayesian posterior probability values at nodes were calculated with a 25% burn-in removed from each run.

### Bayesian concordance analysis

Phylogenetic hypotheses based on concatenated datasets derived from multiple loci may include a mixture of discordant gene trees due to the presence of conflicting genealogical histories [34, 35]. To identify and quantify such phylogenetic discordance in our data set, we performed a Bayesian concordance analysis with loci from the m4 data set using the BUCKy v. 1.4.3 software package [36, 37]. BUCKy takes as input the posterior distribution of trees estimated by a Bayesian phylogenetic analysis of each individual locus, and based on these estimates a primary concordance tree and a concordance factor (CF), which measures the proportion of the loci supporting a given clade [37]. Loci containing at least two parsimony informative sites were extracted from the m4 data set. For each locus, two independent runs were executed in MrBayes v. 3.2.1 with the best-fit nucleotide substitution model selected by jModelTest, each run with four chains for 11.1 million generations sampling every 1000 generations. The posterior distribution of trees from each individual locus was summarized by the program ‘mbsum’ implemented BUCKy with 10% burn-in for each tree file. The summarized tree files of each locus were used as input for BUCKy, in which two independent runs were executed with four chains for 500,000 generations. The α parameter represents the *a priori* level of discordance expected among loci, and we tested two different values for this parameter (0.1 and 100). Since BUCky requires complete data matrices (i.e. no missing data), we had to sacrifice the number of samples to increase the number of loci for the BUCKy analysis and thus only one individual from *D*. *lapponica* and *D*. *obovata* and all individuals from *D*. *purpurea* and *D*. *himalaica* were retained in the BUCKy analysis.

### Testing for admixture using Patterson’s D-statistic test

The four-taxon D-statistic test is based on the assumption of a true four-taxon species tree (((P1, P2) P3,) O). Alleles sampled from these four species will at times suggest phylogenetic patterns that are incongruent with the species tree. Assuming a bi-allelic site composed of alleles ‘A’ and ‘B’, there are two incongruent patterns possible: ABBA and BABA [38, 39]. If stochastic processes such as incomplete lineage sorting are responsible for this incongruence, the two patterns are expected to have equal frequencies, whereas if the incongruence is caused by, for example, introgression between P3 and either P1 or P2, the frequencies ABBA and BABA are expected to be significantly different, and the D-statistic is used to test the significance of this imbalance [39]. In our study, we were interested in testing whether introgression had occurred between *D*. *purpurea* and *D*. *himalaica*, because sequences from those two species were mixed in gene trees based on ITS and plastid DNA presented by Hou *et al*. [27].

All loci from the m4 data set were used in the D-statistic tests, and heterozygous sites were included in the analyses. We had multiple individuals of each species, and thus D-statistic tests were performed using all possible combinations between individuals from the two HHM species. All individuals from *D*. *lapponica* and *D*. *obovata* were used as outgroup (O). In total, 18 tests were conducted, and for each test 1000 bootstrap replicates were performed to measure the standard deviation of the D-statistic. Significance was evaluated by converting the Z-score (which represents the number of standard deviations from zero for D statistic) into a two-tailed P-value, and using α = 0.01 as a conservative cutoff for significance. A significant Z-score (i.e. > 2.55) suggests that gene flow might have occurred between P3 and either P1 or P2.

To visualize the potential admixture between *D*. *purpurea* and *D*. *himalaica*, a network based on the m4 data set excluding the outgroup was constructed using the NeighborNet algorithm implemented in SplitsTree v. 4.13 [40], and a bootstrap analysis was performed with 1,000 replicates.

## Results

### RAD-seq data matrices

After de-multiplexing and quality filtering using pyRAD, the number of reads per sample varied from 0.18 × 10^6^ to 3.11 × 10^6^ with a median value of 0.69 × 10^6^ (Table 1). In a preliminary analysis, the 90% clustering similarity threshold generated the highest number of loci and parsimony informative sites (Fig 2) and was therefore used to construct data sets for further analysis. When clustering reads using the 90% similarity threshold and 2× minimum coverage, the total number of clusters obtained from each sample varied from 1,921 to 17,934 with a median value of 7,650, and the average cluster coverage in each sample varied from 35 to 142 (Table 1). Consensus sequences were called for each cluster in each sample and possible paralogs were filtered out, resulting in 1,787 to 17,392 consensus loci with a median value of 6,815 (Table 1). After blast filtering for contaminants and discarding loci that did not contain parsimony informative sites, the aligned and concatenated m4 data matrix contained 2,650 loci with 51% missing data and a total of 229,949 sites, of which 5,291 (2.30%) were parsimony informative. The aligned and concatenated m14 data matrix contained 114 loci with 21% missing data and a total of 9,870 sites, of which 230 (2.33%) were parsimony informative. BLAST filtering removed 1.38% of the loci from the m4 data set as potential contaminant sequences from metazoa, bacteria or fungi. No potential contaminant sequences were found in the m14 data matrix.

**Table 1:** Results after filtering and clustering RAD-seq data from 14 samples of *Diapensia* and 4 samples of the outgroup using pyRAD.

Species	Sample ID	No. reads (× 10^6^)^a^	Clusters at 90%^b^	Mean depth	No. consensus loci	No. loci^c^		No. of pis^c^	
						m4	m14	m4	m14
*D*. *himalaica* J.D.Hooker & Thomson	55962	0.85	16075	46.91	15579	1853	110	4265	251
	55969	1.14	7954	123.14	6717	1292	103	3035	222
	55976	0.75	7964	84.27	7691	1826	109	4254	244
*D*. *lapponica* L.	43266	0.54	5704	77.77	5529	1647	110	3860	248
	47250	0.63	9966	53.88	9635	1650	109	3864	246
	47410	0.57	11688	41.72	10870	1456	106	3477	242
	55956	1.03	17934	53.68	17392	1936	113	4529	257
*D*. *obovata* (F. Schmidt) Nakai	53658	0.77	7262	92.17	6913	1787	113	4104	257
	55953	1.23	7345	132.56	6568	1496	106	3512	234
	55954	0.38	3589	93.37	3311	650	72	1536	150
	6378	0.61	4957	103.87	4854	1571	109	3601	242
*D*. *purpurea* Diels	56014	1.94	10328	142.37	8632	1699	112	3989	254
	56025	3.11	15991	139.85	13594	2021	114	4645	258
	56031	1.12	11446	88.98	11176	1884	113	4239	254
*Schizocodon soldanelloides* Siebold & Zucc.	56079	0.20	4400	35.46	4124	82	13	199	30
	56080	0.42	4820	80.66	4653	107	12	264	28
*Shortia uniflora* Maxim.	56090	0.36	2865	96.75	2711	257	54	638	136
	56091	0.18	1921	84.10	1787	261	53	626	141

m4/m14: data matrix clustering RAD-seq reads at 90% similarity threshold and consisting of loci that shared by at least 4 (‘m4’) or 14 (‘m14’) samples.

^a^Number of reads after quality filtering.

^b^Clusters that passed filtering for 2× minimum coverage.

^c^After descarding all loci without parsimony informative sites (pis), blast filtering and trimming the last 5 bases from all loci.

### Phylogenetic reconstruction

**Fig 3:**
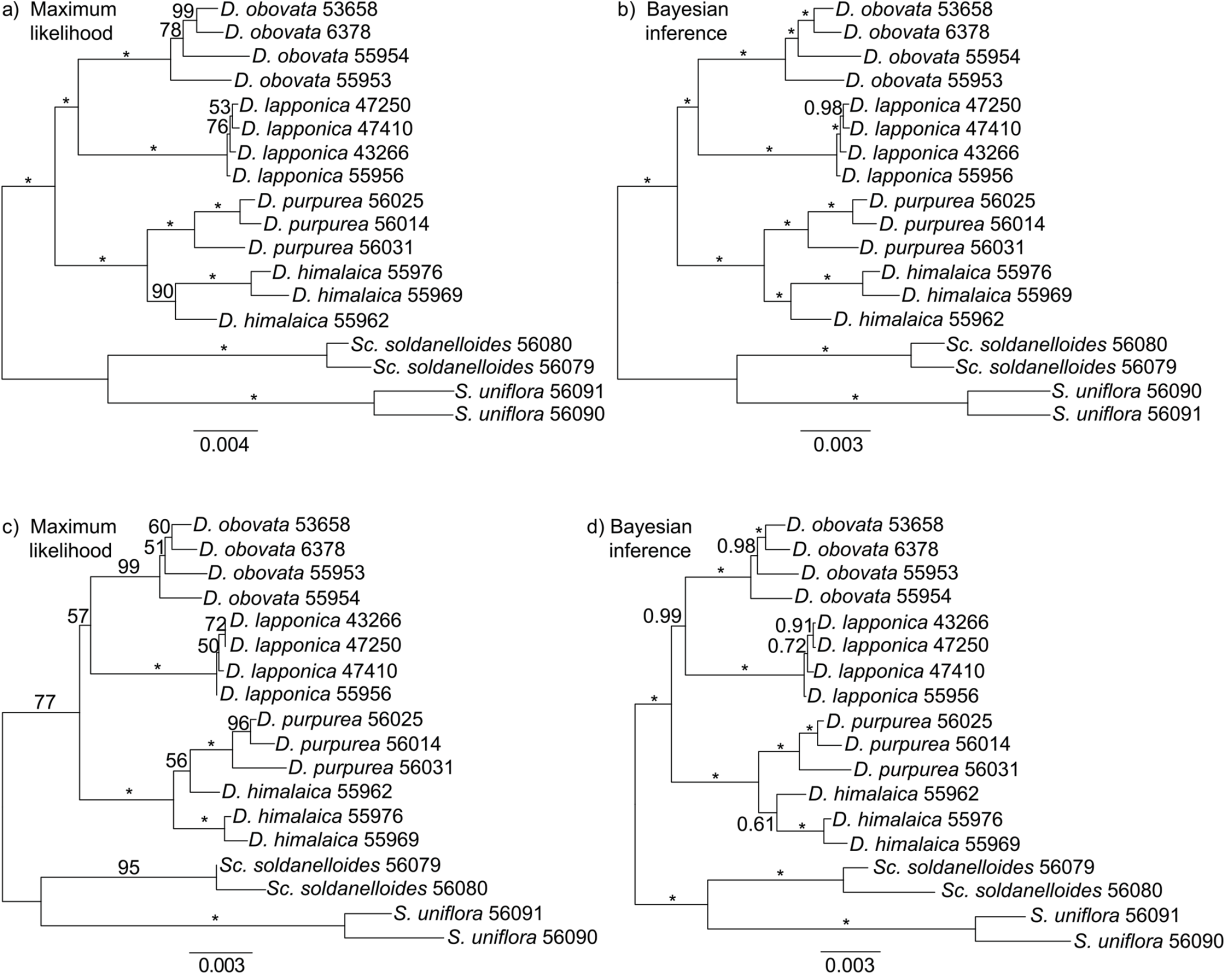
Phylogeny of *Diapensia* inferred from two RAD-seq data sets (a to d). (a) and (b) are based on the m4 data set, in which all loci were shared by at least 4 samples; and (c) and (d) are based on the m14 data set, in which all loci were shared by at least 14 samples. Sample IDs refer to Table 1. Maximum likelihood trees were estimated using RAxML; numbers above branches are bootstrap (BS) values generated from 1,000 replicates. Bayesian consensus trees were inferred using MrBayes; numbers above branches are posterior probabilities (PP). Asterisks on branches indicate BS = 100 or PP = 1.

In the Maximum Likelihood (ML) and Bayesian Inference (BI) analyses based on the m4 data set, *Diapensia* was inferred as monophyletic with the allopatric arctic *D*. *lapponica* and *D*. *obovata* as sisters to the HHM species *D*. *purpurea* and *D*. *himalaica* (Fig 3A and 3B). All species and interspecfic relationships were fully supported both by ML bootstrapping (BS) and Bayesian posterior probabilities (PP). The trees based on the m14 data set were similar except for lower branch support and that *D*. *himalaica* was non-monophyletic (Fig 3C) or very poorly supported as monophyletic (Fig 3D).

### Bayesian concordance analysis

**Fig 4:**
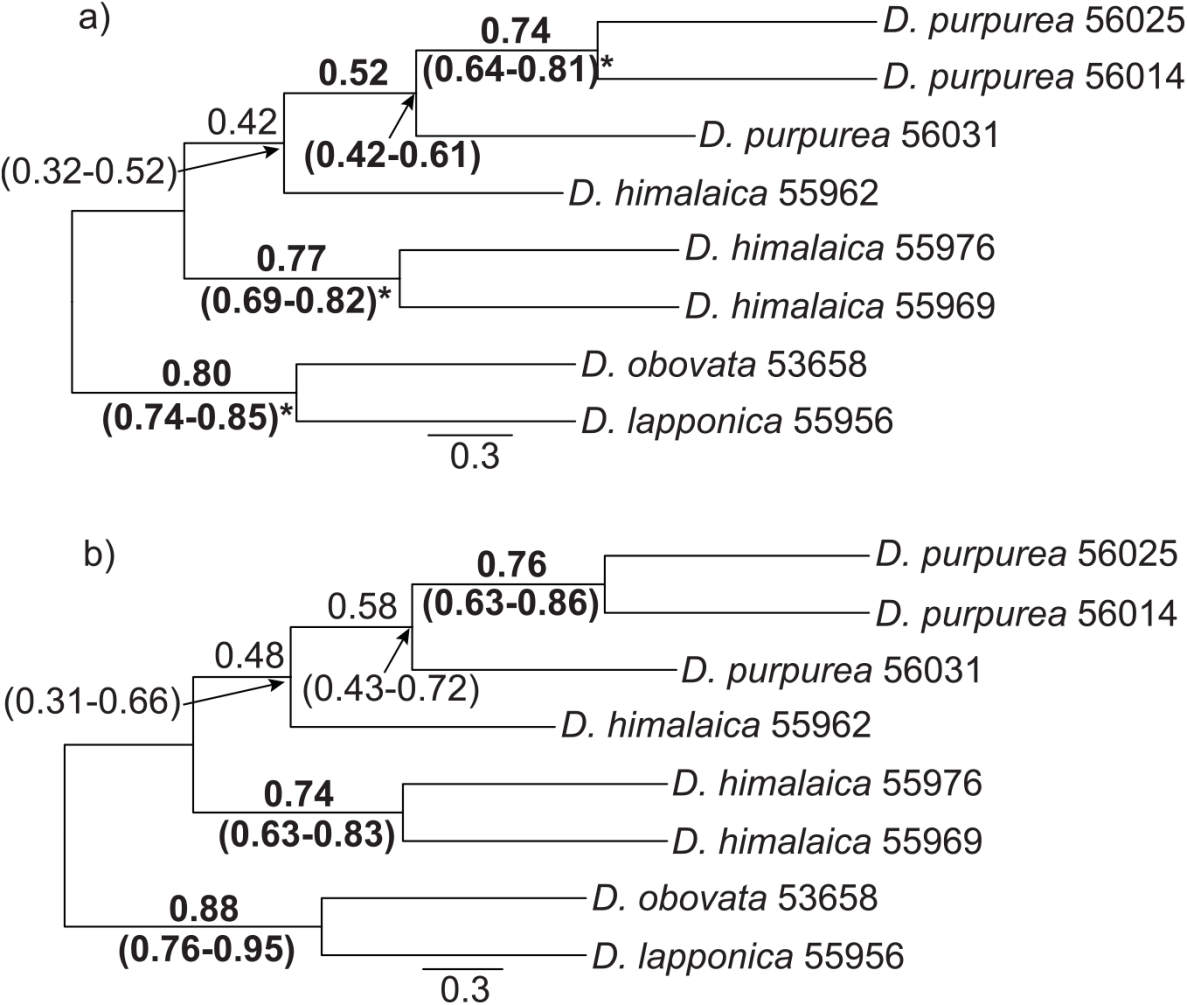
Primary concordance trees inferred at the α of 100 (a) and 0.1 (b) using BUCKy. The α parameter represents the *a priori* level of discordance expected among loci, where a high α assumes a high level of discordance among the gene trees and a low α assumes a low level of discordance. 246 loci with at least two parsimony informative sites covering eight *Diapensia* samples were used in the BUCKy analyses. Sample IDs refer to Table 1. The concordance factors (CFs) and their 95% confidence intervals are shown on the branches; those in bold did not overlap with any conflicting CF, and those in bold and with an asterisk had no conflicting splits.

In the m4 data set, 1,635 loci contained at least two parsimony informative sites. However, because BUCKy requires loci to be shared across all samples we removed several samples to retain more loci for the BUCKy analysis. We kept all *D*. *purpurea*/*D*. *himalaica* samples but chose only one sample each of *D*. *lapponica* and *D*. *obovata* because the two arctic species were fully supported as reciprocally monophyletic in all phylogenetic trees (Fig 3) and have previously been shown to contain very little within species diversity [27]. After excluding loci not shared by all eight *Diapensia* samples (S2 Text), 246 loci were retained for Bayesian concordance analysis. In total, 10,395 different tree topologies and 119 distinct splits were found using BUCKy. The sample-wide mean concordance factors (CFs) and their 95% confidence intervals (CI) are presented on the primary concordance trees (Fig 4). For both primary concordance trees based on different values of the α prior (100 and 0.1), each split was compared with splits that were not present in the primary concordance tree but with estimated CF value above 0.05 (S2 Text). If no conflicting splits were found, or the CI of the conflicting splits did not overlap with the CI of the clade in the primary concordance tree, this clade was considered significantly supported.

The primary concordance trees constructed using different values of the α prior (100 and 0.1) presented the same topology but with different CF supports (Fig 4). For example, the tree using α = 100 significantly supported the *D*. *purpurea* clade (Fig 4A) whereas the tree with α = 0.1 did not (Fig 4B). Whereas the ML and BI analyses based on the m4 data set resolved *D*. *himalaica* as monophyletic (Fig 3A and 3B), the primary concordance trees and the ML and BI analyses based on the m14 data set did not (Figs 4, 3C and 3D).

### Four-taxon D-statistic test for admixture

The ML and BI analyses based on the m14 data set as well as the concordance analysis rejected monophyly for *D*. *himalaica* (Fig 3C and 3D), suggesting conflicting phylogenetic signals. To explore the source of this conflict, we tested for a signal of admixture between *D*. *purpurea* and *D*. *himalaica* using the four-taxon D-statistic test based on the m4 data set (test 1–18 in Table 2). The D-statistics test is reciprocal, and first test if any *D*. *purpurea* sample share more derived alleles with one *D*. *himalaica* sample compared to the other *D*. *himalaica* sample, and then test if any *D*. *himalaica* sample share more derived alleles with one *D*. *purpurea* sample compared to the other *D*. *purpurea* sample. The number of loci available for each test varied from 670 to 1,283, and the percentage of discordant sites ranged from 0.03 to 0.19 (Table 2). Nine out of the 18 tests detected a significant signal of admixture between *D*. *purpurea* and *D*. *himalaica* (test 1–2, 4–8, 11–12; Table 2).

**Table 2:** Four-taxon D-statistic test for introgression between *Diapensia purpurea* and *D*. *himalaica*.

No. test	P1	P2	P3	O	D	Std(D)	Z	ABBA	BABA	No. loci	pdisc
1	him1	him2	pur1	A	-0.58	0.10	5.60*	18.50	69.75	670.00	0.14
2	him1	him3	pur1	A	-0.72	0.08	9.39*	18.75	113.50	980.00	0.15
3	him2	him3	pur1	A	0.17	0.21	0.84	16.00	11.25	690.00	0.04
4	him1	him2	pur2	A	-0.80	0.07	12.25*	11.25	99.25	784.00	0.15
5	him1	him3	pur2	A	-0.76	0.06	13.27*	19.75	142.00	1176.00	0.15
6	him2	him3	pur2	A	0.52	0.19	2.80*	20.50	6.50	814.00	0.03
7	him1	him2	pur3	A	-0.89	0.04	24.38*	7.25	126.00	790.00	0.19
8	him1	him3	pur3	A	-0.88	0.04	24.11*	12.25	187.25	1182.00	0.19
9	him2	him3	pur3	A	0.38	0.22	1.69	13.75	6.25	806.00	0.03
10	pur1	pur2	him1	A	0.01	0.21	0.04	15.50	15.25	1096.00	0.04
11	pur1	pur3	him1	A	0.50	0.08	6.08*	75.00	24.75	1074.00	0.12
12	pur2	pur3	him1	A	0.50	0.08	6.23*	84.50	28.00	1283.00	0.11
13	pur1	pur2	him2	A	-0.16	0.31	0.50	10.25	14.00	771.00	0.03
14	pur1	pur3	him2	A	-0.27	0.24	1.14	9.50	16.50	709.00	0.04
15	pur2	pur3	him2	A	-0.03	0.21	0.16	14.00	15.00	832.00	0.04
16	pur1	pur2	him3	A	-0.14	0.16	0.85	10.25	13.50	1075.00	0.03
17	pur1	pur3	him3	A	0.07	0.21	0.34	17.25	15.00	1034.00	0.03
18	pur2	pur3	him3	A	-0.01	0.17	0.04	20.75	21.00	1242.00	0.04

P1, P2 and P3: him1: *D*. *himalaica* 55962, him2: *D*. *himalaica* 55969, him3: *D*. *himalaica* 55976, pur1: *D*. *purpurea* 56014, pur2: *D*. *purpurea* 56025, pur3: *D*. *purpurea* 56031 (sample IDs refer to Table 1); O: outgroup ‘A’ consists of all individuals from the two arctic species *D*. *lapponica* and *D*. *obovata*. D-statistic values (D) and their standard deviation (Std(D)) are given for each test. ABBA, BABA: the number of alleles that support each pattern (the fractions are due to heterozygosity). No. loci: the number of loci analyzed in each test; pdisc: the percentage of discordance.

*Z-scores that are statistically significant after conversion to a two-tailed P-value and using α = 0.01 as a conservative cutoff for significance.

We also found evidence for admixture between *D*. *purpurea* and *D*. *himalaica* in the network (S1 Fig), showing complicated reticulations between the two species.

## Discussion

### Resolving the deep and shallow histories in *Diapensia*

All our analyses of the RAD-seq data support three main lineages in *Diapensia*, congruent with our previous results from plastid and ITS data [27]. However, whereas the arctic *D*. *lapponica* somewhat surprisingly was resolved as sister to the HHM taxa in the plastid and ITS analysis (see Fig 2 in [27]), our analyses of the RAD-seq data strongly supported *D*. *lapponica* as sister to the arctic *D*. *obovata* (Fig 3). The primary concordance tree, too, supports an arctic and an HHM clade (Fig 4). Where do the conflicting results come from? The age of the *Diapensia* crown group was estimated to Late Miocene and the splits leading to *D*. *lapponica*, *D*. *obovata*, and the HHM clade consisting of *D*. *purpurea* and *D*. *himalaica* were inferred to have occurred approximately at the same time [27]. Although four plastid regions were analyzed by Hou *et al*. [27], they are inherited as a single linkage group and therefore only two loci, one plastid and one nuclear (ITS), were in practice analyzed. Given the small number of loci investigated and the relatively short time separating the two divergences, we suggest that ancient incomplete lineage sorting (ILS) may have played a major role in the discrepancy between the plastid/ITS and RAD-seq phylogenies. We also noticed that a fraction of the RAD-seq loci contained a phylogenetic signal different from the primary concordance tree loci in our concordance analysis (Fig 4), likely caused by ILS. The large number of loci, however, seems to overwhelm the discordant phylogenetic signals in the data. This effect is also clear when comparing the phylogenetic analyses conducted using the two differently sized RAD-seq data matrices, m4 and m14, in which the large data set (m4, 2650 loci) resulted in more strongly supported branches (BS = 100, PP = 1) compared to the small data set (m14, 114 loci; Fig 3).

A similar pattern of “swamping” was seen in the relationships between the HHM species *D*. *himalaica* and *D*. *purpurea*. Both species were supported as monophyletic in the analysis of the large m4 data set (Fig 3A and 3B), but *D*. *himalaica* was non-monophyletic (Fig 3C) or very poorly supported as monophyletic (Fig 3D) in the analysis of the smaller, but more complete m14 data set (Fig 3). Interestingly, the primary concordance tree, based on the m4 data set also rejected monophyly for *D*. *himalaica* (Fig 4). Disregarding technical problems associated with the RAD-seq library preparation procedure, one must invoke biological processes such as admixture (e.g., hybridization and introgression), ILS, and “hidden paralogy” due to gene duplications and losses, to explain these patterns of phylogenetic conflict [41]. Although paralogy due to gene duplications and losses may pose a significant problem [42, 43], it is unlikely that it would be frequent enough to be a major source of incongruence in a recently [27] diverged group such as *D*. *himalaica* and *D*. *purpurea*. Thus, the two remaining plausible causes of the conflicting signals are admixture and ILS. The D-statistic test detected nine instances of significant gene flow between *D*. *purpurea* and *D*. *himalaica* (test 1–2, 4–8, 11–12; Table 2), seven of which showed admixture between *D*. *himalaica* sample 55962 and any sample of *D*. *purpurea*. One test detected a significant signal for admixture between *D*. *himalaica* sample 55976 and *D*. *purpurea* sample 56025, relative to the *D*. *himalaica* sample 55969 (test 6; Table 2). Interestingly, these two *D*. *himalaica* samples are from the same locality, approximately 300 km from the *D*. *purpurea* sample 56025 (Fig 1).

Given that *D*. *purpurea* was supported as monophyletic in all analyses (Fig 3), and the D-statistic tests only suggested admixture between one sample of *D*. *himalaica* and *D*. *purpurea*, the direction of the gene flow is likely from *D*. *purpurea* to this particular sample of *D*. *himalaica*. However, we speculate that more samples of *D*. *himalaica* and *D*. *purpurea* were involved and that the gene flow may be reciprocal as the ITS gene tree rejected monophyly for both *D*. *himalaica* and *D*. *purpurea* as multiple accessions of the two species were intermixed [27]. A more extensive sampling of *D*. *purpurea* and *D*. *himalaica*, particularly in regions of sympatry, will be key to future studies of the dynamic history of these two species.

### Performance of RAD-seq for interspecific phylogenetic reconstructions

In this study, we used the pyRAD pipeline to construct two RAD-seq data matrices that vary in the amount of missing data but with otherwise identical parameter settings: the larger and thus less complete m4 data matrix, and the smaller and thus more complete m14 data matrix. The m14 data set contained significantly less missing data (21% missing data compared to 51%) and fewer parsimony informative sites (230 sites compared to 5,291) compared to the m4 data set. Nevertheless, the tree topologies based on the two data sets are very similar (Fig 3). This result is consistent with the study by Rubin *et al*. [16] where they reconstructed phylogenies using simulated RAD-seq data matrices with missing data ranging from 6% to 67%, and concluded that large amounts of missing data in RAD-seq data matrices did not adversely affect the accuracy of phylogenetic inference. Although our two analyses resulted in similar tree topologies, full support and monophyly of all species was obtained only for the m4 data set (Fig 3), which contained many more parsimony informative sites. Similar results have been obtained in studies of *Pedicularis* [18] and cichlid fishes [22], which are clades well known for hybridization and introgression. In both of these studies data sets that varied in their degree of “missingness” also resulted in similar tree topologies but the largest, and thus most informative, data matrices resulted in the highest phylogenetic support.

The analysis of the m4 data matrix consisting of 229,949 sites and 51% missing data, fully resolved two deep divergences that are nearly temporally coincident in the Late Miocene [27], as well as a more recent Pleistocene divergence, which may be obscured by some signal of admixture (Fig 3A and 3B). Our results add to the growing number of studies suggesting that RAD-seq is a simple and cost-effective way of generating large amounts of genome-wide phylogenetic markers suitable for inferring interspecific phylogenies without previous assembly of genomic resources.

## Supporting Information

S1 Fig NeighborNet constructed by Splitstree using equal-angle split transformation, based on the m4 data set excluding the outgroup. All edges have 100% bootstrap support.
(EPS)

S1 Table Collecting information of samples analyzed in this study. (XLSX)

S2 Table Results of jModelTest. (XLSX)

S1 Text Protocol for preparing the RAD-seq libraries. (DOCX)

S2 Text Results of Bayesian concordance analysis in BUCKy at two values for the prior alpha. (DOCX)
