# Supplementary material for: Thousands of RAD-seq Loci Fully Resolve the Phylogeny of the Highly Disjunct Arctic-Alpine Genus Diapensia (Diapensiaceae)
Source: PLoS One. 2015 Oct 8;10(10):e0140175. doi: 10.1371/journal.pone.0140175 (PMC4598014; doi:10.1371/journal.pone.0140175)
Supplement: S1 Text — (DOCX) [file pone.0140175.s004.docx]

**S2. Protocol for preparing the RAD-seq libraries**

The amounts of DNA in all extractions were measured using Qubit fluorometer (Invitrogen, Carlsbad, CA, USA). In those cases when the DNA concentration was lower than 6.6-10 ng/µL or when there was too little DNA left, a whole genome amplification (WGA) was performed using the REPLI-g Kit (Qiagen, Valencia, CA, USA) following the manufacturer's instructions.

Genomic DNA (200-300 ng) was digested for 90 min at 37°C in a 50 µL reaction with 5 µL 10× CutSmart Buffer (New England Biolabs [NEB], Ipswich, MA, USA), 10 units (U) *Sbf*I High Fidelity Restriction Enzyme (NEB) and 14.5 µL H_2_O, followed by a heat inactivation of 20 min at 80°C.

2.5 µL of 100 nM P1 Adaptor, a modified Solexa© adapter (2006 Illumina, Inc.; for *Sbf*I digestion, top: 5’-AATGATACGGCGACCACCGAGATCTACACTCTTTCCCTACACGACGCTCTTCCGATCTxxxxTGCA-3’, bottom: 5’-Phos-xxxxAGATCGGAAGAGCGTCGTGTAGGGAAAGAGTGTAGATCTCGGTGGTCGCCGTATCATT-3’ [x = barcode]) was added to the digested samples along with 0.6 µL 100 mM rATP (Promega, Fitchburg, WI, USA), 1 µL 10× CutSmart Buffer, 1000 U T4 DNA ligase (high concentration, NEB), and 5.4 µL H_2_O and incubated at room temperature for 30 min. This was followed by a heat inactivation of 20 min at 65°C.

The barcoded samples were pooled and the DNA randomly sheared using a Bioruptor (Diagenode, Denville, NJ, USA). Shearing was done in volumes of 240-300 µL per tube containing 860-1200 ng DNA each using 7 cycles of 30 sec on/ 30 sec off, at high.

The sheared samples were combined to a total of 10 µL and run on a 1.5% agarose, 0.5× TBE gel to check for the presence of a smear in the 100-700 bp range. The combined samples were purified with QIAquick PCR Purification Kit Columns (Qiagen) following the manufacturer's instructions and eluted in 21 µL elution buffer.

The purified samples were run on a 1.5% agarose 0.5× TBE gel, and the smear between 250-500 bp was cut out. DNA was recovered using MinElute Gel Extraction Kit (Qiagen) following manufacturer’s instructions except for an initial 20 min incubation at 24°C with agitation and a final elution with 20 µL elution buffer in a tube already containing 2.5 µL blunting buffer (NEB).

We added 2.5 µL of 1mM dNTP mix and 1 µL Blunt Enzyme Mix (Quick Blunting Kit; NEB) to the eluate from the previous step and incubated 30 min at room temperature. The sample was purified using Quick Spin Columns (Qiagen) following the manufacturer’s protocol. The final elution was obtained with 20 µL elution buffer in a tube already containing 5 µL NEB buffer 2 (NEB).

To this eluate we added 1 µL 10mM dATP (Invitrogen) and 15 U of Klenow fragment (NEB) and incubated at 37°C for 30 min and let to slowly cool to room temperature.

The samples were then purified using Agencourt AMPure XP beads (Beckman Coulter, Krefeld, Germany) in the ratio of 0.8 XP beads/sample volume but otherwise following the manufacturer’s instructions. The final elution was done with 45 µL elution buffer (Qiagen) and the eluate transferred to a new tube containing 5.3 µL NEB buffer 2 (NEB).

To this eluate we added 1 µL of 10 µM P2 Adapter, a modified Solexa© adapter (2006 Illumina, Inc.; top: 5’-Phos-CTCAGGCATCACTCGATTCCTCCGAGAACAA-​3’, bottom: 5’-CAAGCAGAAGACGGCATACGACGGAGGAATCGAGTGA​TGCCTGAGT-3’), 0.5 µL 100mM rATP (Promega) and 1000 U T4 Ligase (high concentration, NEB), and incubated it at room temperature for 30 min.

The sample was again purified with AMPure XP beads and eluted in 52 µL elution buffer (Qiagen). 4 µL of this product was used in a preliminary PCR amplification with 12.5 µL Phusion High-Fidelity Master Mix (NEB), 1 µL of each 10 µM modified Solexa© Amplification primer mix (2006 Illumina, Inc.; P1-forward primer: 5’-AATGATACGGCGACCACCGA-3’; P2-reverse primer: 5’-CAAGCAGAAGACGGCATACGA-3’), and 6.5 µL H_2_O to a total volume of 25 µL. PCR conditions were: 30 sec at 98°C followed by 23 cycles of 10 sec at 98°C, 30 sec at 65°C, and 30 sec at 72°C, ending with a final 5 min extension at 72°C. After checking the PCR result on a 1.5% agarose 0.5× TBE gel, the remaining purified products were amplified in a 100 µL reaction using the same protocol. The reaction was distributed into 8 tubes with 12.5 µL each in order to minimize any bias in the PCR reaction. 5 µL of the pooled PCR product were run on a 1.5% agarose 0.5× TBE gel together with 1 µL of the template library. Successful libraries resulted in a band in the 500 bp range, about 10 times stronger and with a tighter length distribution than the template library. The amplified library was then purified with AMPure XP beads with a final elution in 30 µL elution buffer.

The DNA concentration of the purified library was measured with a Qubit fluorometer (Invitrogen, Carlsbad, CA, USA), the salt concentration was estimated with a Nanodrop ND-1000 spectrophotometer (Nanodrop Technologies, Wilmington, DE, USA), and ran on an Agilent 2100 Bioanalyzer chip (Agilent technologies, Santa Clara, CA, USA) to visualize the fragment size distribution. An average of 12-14 ng/ul was obtained for almost all libraries. The libraries were sequenced at the Norwegian Sequencing Centre (<http://www.sequencing.uio.no>) on an Illumina HiSeq 2000 instrument using 101 cycles of paired end sequencing.
