# Supplementary material for: Thousands of RAD-seq Loci Fully Resolve the Phylogeny of the Highly Disjunct Arctic-Alpine Genus Diapensia (Diapensiaceae)
Source: PLoS One. 2015 Oct 8;10(10):e0140175. doi: 10.1371/journal.pone.0140175 (PMC4598014; doi:10.1371/journal.pone.0140175)
Supplement: S2 Text — (DOCX) [file pone.0140175.s005.docx]

**S4.** **Results of Bayesian concordance analysis in BUCKy at two values for the prior alpha**

Alpha = 100

translate

1 *D. himalaica* 55962,

2 *D. himalaica* 55969,

3 *D. himalaica* 55976,

4 *D. lapponica* 55956,

5 *D. obovata* 53658,

6 *D. purpurea* 56014,

7 *D. purpurea* 56025,

8 *D. purpurea* 56031;

Population Tree:

((1,((2,3),(4,5))),(6,7),8);

Primary Concordance Tree Topology:

((1,((2,3),(4,5))),(6,7),8);

Population Tree, With Branch Lengths In Estimated Coalescent Units:

((1:10.000,((2:10.000,3:10.000):1.378,(4:10.000,5:10.000):1.508):0.317):0.514,(6:10.000,7:10.000):1.232,8:10.000);

Primary Concordance Tree with Sample Concordance Factors:

((1:1.000,((2:1.000,3:1.000):0.765,(4:1.000,5:1.000):0.797):0.422):0.518,(6:1.000,7:1.000):0.740,8:1.000);

Four-way partitions in the Population Tree: sample-wide CF, coalescent units and Ties(if present)

{1,2,3,4,5; 8|6; 7} 0.806, 1.232,

{1; 2,3,4,5|6,7; 8} 0.601, 0.514,

{1,6,7,8; 4,5|2; 3} 0.832, 1.378,

{1,6,7,8; 2,3|4; 5} 0.852, 1.508,

{1; 6,7,8|2,3; 4,5} 0.514, 0.317,

Splits in the Primary Concordance Tree: sample-wide and genome-wide mean CF (95% credibility), SD of mean sample-wide CF across runs

{1,2,3,6,7,8|4,5} 0.797(0.744,0.846) 0.593(0.529,0.656) 0.002

{1,4,5,6,7,8|2,3} 0.765(0.691,0.821) 0.570(0.498,0.636) 0.002

{1,2,3,4,5,8|6,7} 0.740(0.642,0.805) 0.553(0.469,0.624) 0.006

{1,2,3,4,5|6,7,8} 0.518(0.415,0.610) 0.377(0.289,0.460) 0.004

{1,6,7,8|2,3,4,5} 0.422(0.317,0.516) 0.307(0.221,0.392) 0.003

Splits NOT in the Primary Concordance Tree but with estimated CF > 0.050:

{1,2,3|4,5,6,7,8} 0.317(0.232,0.411) 0.234(0.160,0.316) 0.004

{1,8|2,3,4,5,6,7} 0.238(0.154,0.337) 0.195(0.124,0.279) 0.006

Alpha = 0.1

translate

1 *D. himalaica* 55962,

2 *D. himalaica* 55969,

3 *D. himalaica* 55976,

4 *D. lapponica* 55956,

5 *D. obovata* 53658,

6 *D. purpurea* 56014,

7 *D. purpurea* 56025,

8 *D. purpurea* 56031;

Population Tree:

((1,((2,3),(4,5))),(6,7),8);

Primary Concordance Tree Topology:

((1,((2,3),(4,5))),(6,7),8);

Population Tree, With Branch Lengths In Estimated Coalescent Units:

((1:10.000,((2:10.000,3:10.000):1.649,(4:10.000,5:10.000):1.967):0.423):0.529,(6:10.000,7:10.000):1.278,8:10.000);

Primary Concordance Tree with Sample Concordance Factors:

((1:1.000,((2:1.000,3:1.000):0.744,(4:1.000,5:1.000):0.882):0.475):0.579,(6:1.000,7:1.000):0.761,8:1.000);

Four-way partitions in the Population Tree: sample-wide CF, coalescent units and Ties(if present)

{1,2,3,4,5; 8|6; 7} 0.814, 1.278,

{1; 2,3,4,5|6,7; 8} 0.607, 0.529,

{1,6,7,8; 4,5|2; 3} 0.872, 1.649,

{1,6,7,8; 2,3|4; 5} 0.907, 1.967,

{1; 6,7,8|2,3; 4,5} 0.563, 0.423,

Splits in the Primary Concordance Tree: sample-wide and genome-wide mean CF (95% credibility), SD of mean sample-wide CF across runs

{1,2,3,6,7,8|4,5} 0.882(0.764,0.951) 0.882(0.753,0.959) 0.039

{1,2,3,4,5,8|6,7} 0.761(0.626,0.858) 0.761(0.617,0.869) 0.044

{1,4,5,6,7,8|2,3} 0.744(0.626,0.833) 0.744(0.610,0.848) 0.047

{1,2,3,4,5|6,7,8} 0.579(0.431,0.720) 0.579(0.418,0.733) 0.071

{1,6,7,8|2,3,4,5} 0.475(0.305,0.654) 0.475(0.294,0.668) 0.093

Splits NOT in the Primary Concordance Tree but with estimated CF > 0.050:

{1,2,3|4,5,6,7,8} 0.337(0.191,0.455) 0.337(0.180,0.475) 0.064

{1,8|2,3,4,5,6,7} 0.290(0.126,0.459) 0.290(0.117,0.473) 0.081

{1,2|3,4,5,6,7,8} 0.129(0.012,0.260) 0.129(0.006,0.274) 0.063

{1,2,3,4,5,6|7,8} 0.075(0.012,0.183) 0.075(0.005,0.194) 0.036

{1,4,5,8|2,3,6,7} 0.065(0.020,0.126) 0.065(0.017,0.134) 0.013

{1,6,7|2,3,4,5,8} 0.064(0.028,0.118) 0.064(0.019,0.130) 0.011

{1,7,8|2,3,4,5,6} 0.059(0.012,0.142) 0.059(0.010,0.152) 0.025

{1,2,3,5,6,7|4,8} 0.052(0.000,0.106) 0.052(0.000,0.119) 0.015
